# Book Reviews

**Published:** 1902-01

**Authors:** 


### BOOK REVIEWS.

THE DIAGNOSTICS OF INTERNAL MEDICINE. A Clinical Treatise upon the Recognised Principles of Medical Diagnosis. Prepared for the Use of Students and Practitioners of Medicine. By GLENTWORTH REEVE BUTLER, A. M., M. D., chief of the second medical division, Methodist Episcopal Hospital; Attending Physician to the Brooklyn Hospital, etc. Octavo, pp. 1077. With 5 colored plates and 246 illustrations and charts in the text. New York: D. Appleton & Co. 1901.

This book is a treatise upon a subject that concerns every physician who would keep himself well informed in the science and art of diagnosis. A useful preliminary is a well-arranged schedule of examinations with which the book begins, and then follow a few pages of preliminary considerations which include instructions on recording cases. Then the treatise proper commences and it is divided into two parts.

In the first part the evidences of disease are discussed at length and in elaborate detail, 44 sections, comprising nearly 650 pages, being assigned to their consideration. Almost every condition or item that can throw any light upon, or lead to the discovery of any existing disease in the human economy, is herein set forth. History, posture, gait, general appearance, temperament, dress, temperature, condition of skin, digestion, special senses, blood, the cavities, viscera, genito-urinary system, sputum, excretions —these and all other organs, tissues or states of the system are made to yield any evidence they possess, under the searching eye and hand of this author.

The second part is given over to the consideration of direct and differential diagnosis. In this Butler presents 11 sections, covering about 375 pages, in which he discusses the infectious diseases, diseases of the digestive system, of the respiratory system, of the circulatory system, of the blood and ductless glands, of the kidney, of the nervous system, of the muscles, constitutional diseases, the intoxications, sunstroke, and the diseases due to animal parasites. He gives succinct descriptions of these several diseases, together with their usual symptoms, and then applies the principles of diagnosis, direct and differential, in each disease.

These two parts complement each other and unite to make a symmetrical whole in the most satisfactory manner. The book contains everything of practical value relating to diagnosis and represents a vast amount of original work, put together in a most artistic and scientific manner. It contains much material nowhere else to be found and, besides, in it is sorted and grouped everything of value to be found elsewhere that could possibly aid in making a correct diagnosis.

The illustrations deserve special mention. The regions of the trunk have been clearly mapped upon nude figures, male and female, and the location of viscera are shown in diagram and drawing to a considerable extent. The ingenuity of the artist has been cleverly blended with science, leaving nothing to be desired but much to commend in the pictorial part of the work.

There are some abominations in orthography that should be corrected in future editions. For example, "colour" and "labourer" belong to a past not consistent with the immediate present with which the work scientifically teems.

---

Diseases of the Intestines. By Dr. I. Boas, Specialist for Gastro-intestinal Diseases in Berlin. Authorised translation from the first German edition, with special additions by Seymour Basch, M. D., New York City. Octavo, pp. 574, with 47 illustrations. New York: D. Appleton & Co. 1901. [Price, cloth, $5.00; sheep, $6.00.]

Knowledge of diseases of the chylopoietic system has increased very much of late, a fact due very largely to the effective and ingenious work of the author of this book. His treatise became popular in Germany, and its translation into English is in direct response to a demand for an exhaustive work on this branch of medicine in that tongue.

As might be anticipated the author pays great attention to the pathological aspect of his subject and also to the physiologico-chemical view. He gives minute directions as to the examination of the patient, and presents an exhaustive chapter on the examination of the feces. This method of diagnosis is too often neglected or indifferently applied. It is, according to this author, an integral factor in the diagnosis of intestinal diseases. It is to be hoped that what Boas has written will stimulate the more effective application of this important method. Short chapters set forth, also, the diagnostic value of examinations of the stomach contents and urine.

In the chapter allotted to the consideration of the therapeutics of intestinal diseases this author is clear and presents an amount of material that deserves careful study. In a special division the most important intestinal diseases are considered in a manner that will prove of great aid to the clinical management of these affections. Boas acknowledges a great indebtedness to surgery for the contribution it has made toward the solution of many problems relating to diseases of the intestinal tract, especially as regards appendicitis, intestinal obstruction, and tumors. An index giving a list of subjects and a list of authors closes this valuable treatise.

---

Progressive Medicine. Volume III., September, 1901. A quarterly digest of advances, discoveries and improvements in the medical and surgical sciences. Edited by Hobart Amory Hare, M. D., Professor of Therapeutics and Materia Medica in the Jefferson Medical College of Philadelphia. Octavo, 428 pages, 16 illustrations. Philadelphia and New York: Lea Brothers & Co. [Per annum, in four cloth-bound volumes, $10.00.]

The departments considered in this volume are: (1) Diseases of the thorax and its viscera, including the heart, lungs and bloodvessels, by William Ewart (of London); (2) dermatology and syphilis, by William S. Gottheil; (3) diseases of the nervous system, by William G. Spiller; (4) obstetrics, by Richard C. Norris.

Recent studies have advanced our knowledge of diseases of the respiratory tract in a marked degree and Ewart has presented in his article the essentials of everything pertaining to his department. Gottheil, too, has grouped all the recent information relating to dermatology and syphilis, including phototherapy and the Fensen light treatment; also he discusses blastomycosis and ioculation tuberculosis.

In the section on nervous diseases Spiller presents the newest points relating to tumors and abscesses of the brain, besides much other valuable material. In obstetrics Norris sets forth the question of toxins in strong light in discussing eclampsia, and many other obstetrical questions of importance are considered. Viewed as a whole this is one of the most interesting books in this very interesting series.

---

A SYSTEM OF PHYSIOLOGIC THERAPEUTICS. A practical exposition of the methods, other than drug-giving, useful in the prevention of disease and in the treatment of the sick. Edited by SOLOMON SOLIS COHEN, A. M., M. D., Professor of Medicine and Therapeutics in the Philadelphia Polyclinic; Lecturer on Clinical Medicine at Jefferson Medical College; Physician to the Philadelphia Hospital, etc. In eleven 8vo volumes. Volumes III.–IV. Climatology: Health Resorts, Mineral Springs. By F. Parkes Weber, M.A., M. D., F. R. C. P. (Lond.), Physician to the German Hospital, Dalston; Assistant Physician North London Hospital for Consumption, etc. With the collaboration for America of Guy Hinsdale, A. M, M. D., Secretary of the American Climatological Association, etc. In two books. Book I.—Principles of Climatotherapy, Ocean Voyages, Mediterranean, European and British Health Resorts. Book II.—Mineral Springs, Therapeutics, etc. Illustrated with maps, etc. Philadelphia: P. Blakiston's Son & Co. 1901. [Price for the complete set, $22.00 net.]

In these two volumes will be found everything important to know concerning climate, health resorts, mineral springs, and sanitoriums for special diseases, as applied to the management of those maladies that are amenable to such environments. These are conditions too little understood, or indifferently applied in the treatment of diseases, or in its prevention.

In volume three the principles of climato-therapy are discussed, which includes the physics, physiology, and general therapeutics of climate, to the consideration of which the first part is allotted. The description of health resorts makes up the second part, which runs through the remainder of this volume, and also is continued in the fourth volume. Then climato-therapeutics are taken up, forming part three, and this subject continues to the end of the fourth volume. Each volume concludes with an elaborate index, more complete in its detailed information than anything of the kind we have yet seen. The chapter on ocean voyages is of special interest and contains information of greatest value not to be found elsewhere.

The treatment of all the subjects in these two volumes is strictly scientific in character and the information they contain will prove of great aid to every practising physician.

PRACTICAL SURGERY. A Work for the General Practitioner. By NICHOLAS SENN, M. D , Ph. D., LL. D., Professor of Surgery, Rush Medical College, Chicago. Octavo volume, 1133 pages, with 650 illustrations, many in colors. Philadelphia and London: W. B. Saunders & Co. 1901. [Price, cloth, $6 00 net.]

The author announces that this work is not intended to cover the whole surgical field, but only such parts as are of practical importance to the general practitioner of medicine. This is as it should be, for the domain is too great to be discussed in detail and in its entirety in one book by a single author without making an unwieldy volume. There are parts of it, too, which belong almost exclusively to the practitioner of surgery. He must do the surgery of deliberation, the surgery of chronic disease, while the general practitioner must be prepared for the surgery of emergency. This is what Senn's book proposes to do for him.

The author has given very complete directions regarding local and general anesthesia—matters of the utmost importance in such a work; for hemostasis; for the treatment of hemorrhage; for asepsis and antisepsis; for drainage and for suturing and other dressings. These all deserve careful study by those for whom the book is specifically intended. Fractures and dislocations occupy more than a quarter of the book. These are also important subjects to the general practitioner, for they constitute a large proportion of his surgical work; particularly so with regard to fractures. The Röntgen rays have contributed largely toward the illustrations in this portion of the work and some of the plates are very striking in delineating fractures of the long bones. Under the head of "special fractures" Senn describes fractures of the neck of the femur, Colles's fracture and fractures of the skull. This is a very interesting chapter.

A miscellaneous list of subjects is next considered, such as exploratory puncture, subcutaneous medication, paracentesis and drainage of suppurating joints. Then comes aseptic catheterisation, emergency operations upon the air passages and empyemas. After a lengthy chapter on peritonitis he comes to the consideration of appendicitis. Senn is most interesting in his discussion of this subject. It is essentially a surgical disease and he handles it in a surgical manner. In the chapters upon intestinal surgery a masterful hand is observed. He discusses tuberculosis with great scientific acumen, and teaches many valuable ideas.

Resection of joints is one of the most important features of modern surgery, an often-to-be-employed substitute for amputation, by which a useful member is often saved. Its establishment as an acknowledged procedure is one of the triumphs of latter-day surgery. Amputations and disarticulations is the closing chapter, and then follows a well prepared index. The illustrations are copious, sometimes excellent, and always helpful.

Senn is a hardworking student of his art and is a pathologist of no mean accomplishments. In some places this work shows evidence of hasty preparation, but as an intelligent exposition of the science of surgery as practised today it is deserving of commendation, and it will be particularly welcomed by the general practitioner of medicine for whom it is specially prepared. The distinguished author has contributed many books of value to the literature of medicine, and this one is by no means the least important of them.

---

OPERATIVE SURGERY. By JOSEPH D. BRYANT, M.D., Professor of the Principles and Practice of Surgery, Operative and Clinical Surgery, University and Bellevue Hospital Medical College; Visiting Surgeon to Bellevue and St. Vincent's Hospital, etc. Vol. II. Operations on Mouth, Nose and Esophagus, the Viscera connected with the Peritoneum, the Thorax and Neck, Scrotum and Penis, and miscellaneous operations. Octavo, pp. 729. 857 illustrations, including 40 which are colored. New York: D. Appleton & Co. 1901.

One of the attractions of this work is its practical character. In it almost every injury and operation that the everyday practitioner is likely to meet is described and the majority of them are illustrated. This gives the treatise a strong hold upon the general physician who, in the country at least, must be a surgeon. The surgery of special regions, dealt with in this volume, has seldom been delineated with as much clearness either in text or picture. Take, for example, the operations on the liver, and we fail to see how even the novitiate could fail to comprehend the descriptions and illustrations presented by this author. Of course, the specialist will need something more and in greater detail, but here the junior practitioner will find all-sufficient for his earlier needs. And, besides, the information is based upon the soundest surgical principles.

One of the marvels of the work is the profuseness of illustration that everywhere meets the eye. The engraver's art has been employed with a lavishness that provokes wonder and admiration, and with a degree of accuracy that, for the most part, affords satisfaction. We commend the entire work to every man interested in surgery.

---

THE HYGIENE OF TRANSMISSIBLE DISEASES: Their Causation, Modes of Dissemination and Methods of Prevention. By A. C. ABBOTT, M. D., Professor of Hygiene and Bacteriology, University of Pennsylvania. Second edition, revised and enlarged. Octavo, 351 pages, with numerous illustrations. Philadelphia and London: W. B. Saunders & Co. 1901. [Price, cloth, $2.50 net.]

The first edition of this book appeared about two years ago and was accorded an appreciative welcome. Its field had never yet been attempted by any other writer and the edition promptly became exhausted. The preparation of this second edition has enabled the author to modify opinions or amplify them, as the case may be. Some new light lately has been thrown upon the role of mosquitos in spreading yellow fever which had been discussed in this book, but in view of the demonstrations of Gorgas, at Havana, it is probable that this insect hereafter will be accorded a much larger place as a disseminator of this disease.

The author has enlarged and carefully revised the sections on malaria, yellow fever, plague, filariasis, dysentery, and tuberculosis, bringing the views expressed forward to include recent observations and investigations, concerning these maladies. He also has given color to the part rats may play in carrying the contagion of bubonic plague—a view now held by many observers. It is a book that should be in the hands of every enlightened physician, and particularly health officers of large cities should give it careful study.

---

THE ILLINOIS STATE BOARD OF HEALTH. Report of the Sanitary Investigations of the Illinois river and its tributaries. With special reference to the effect of the sewage of Chicago on the Des Plaines and Illinois rivers prior to, and after, the opening of the Chicago drainage canal. J. A. EGAN, M. D., Secretary.

This report possesses special interest at this time in view of the construction of the Chicago sewage çanal, by which it is contemplated turning the entire sewage of that great city into the Des Plaines and Illinois rivers. It is highly important that the state shall exercise complete sanitary supervision of all public water supplies and sewerage systems, and a study of this report by sanitary engineers and public health officials will prove of great interest in relation to the aforementioned problem.

---

A REFERENCE HANDBOOK OF THE MEDICAL SCIENCES. Embracing the Entire Range of Scientific and Practical Medicine and Allied Science. By various writers. A new edition, completely revised and rewritten. Edited by ALBERT H. BUCK, M. D., New York City. Volume III. CHL-EQU. Illustrated by chromo-lithographs and 676 half-tone and wood engravings. Nine volumes, imperial 8vo. New York: William Wood & Co. 1901. [Price, muslin, $6.00 per volume; leather, $7.00 per volume; half-morocco, $8 00 per volume.]

The list of contributors to this volume of this great work is a long one and includes the names of some of the most eminent men in the profession. Drs. Julius Pohlman and Grover W. Wende are the contributors from Buffalo. Many of the articles are almost exhaustive in character. Those on the chorion and the circulation of the blood are nearly if not quite so.

The other articles worthy of mention are so numerous that it would require pages of the JOURNAL to print them, but attention may be called to one on dietetics of the sick as worthy of note, and Koller's article on cocaine possesses special interest in view of the author's discoveries and investigations relating to the physiological action of the drug, especially as applied to ophthalmology.

The illustrations are copious and excellent, some of them, indeed, are superb, plate xxiii. showing affections of the cornea, and xxv. showing the humor tympanic membrane in health and disease, being of the latter order. Such a work represents an enormous outlay of time and money and it is a distinct aid to the study of the subjects it presents.

The American Illustrated Medical Dictionary. For Practitioners and Students. A Complete Dictionary of the Terms used in Medicine, Surgery, Dentistry, Pharmacy, Chemistry, and the kindred branches, including much collateral information of an encyclopedic character, together with new and elaborate tables of Arteries, Muscles, Nerves, Veins, etc; of Bacilli, Bacteria, Micrococci, Streptococci; Eponymic Tables of Diseases, Operations, Signs and Symptoms, Stains, Tests, Methods of Treatment, etc., etc. By W. A. Newman Dorland, A. M., M. D., editor of the "American Pocket Medical Dictionary." Second edition, revised. Octavo, 770 pages, flexible leather. Philadelphia and London: W. B. Saunders & Co. 1901. [Price, $4.50 net.]

Within the past few years a number of good dictionaries have been placed on the market, each having some special claims to excellence, and all being worthy of praise. This one is intended to occupy a medium position between the large unabridged dictionary and the condensed students' edition. Its orthography is modern, its definitions are clear even if concise, and its pronunciation is beyond criticism. Its letter-press is clear and handsome, its tables are well prepared, and its illustrations are excellent, its arrangement convenient and its binding in flexible red leather is attractive. Its corners being rounded serve to preserve them from wear and tear and its red edges add to its general beauty.

These are a few of its distinctive qualities that go to make up one of the most acceptable dictionaries of its class. The first edition, a large one, was exhausted within eight months and this new one will be welcomed by a large number of purchasers without doubt. We have seldom seen a lexicon that equalled it in its class, and we know of none superior. The author and publisher deserve unstinted praise for the parts each have played in making such a superb and beautiful book.

---

The Principles and Practice of Medicine. Designed for the use of Practitioners and Students. By Wm. Osler, M. D., Fellow of the Royal Society; Fellow of the Royal College of Physicians, London; Professor of Medicine in the Johns Hopkins University and Physician-in-Chief to the Johns Hopkins Hospital, Baltimore, etc. Octavo, pp. 1200. Fourth edition. New York: D. Appleton & Co. 1901. [Price, cloth, $5.50; sheep, $6.50; half-morocco, $7.00.]

From the first this work took a high position and has become a favorite as a text-book on practice. In diagnosis and pathology it is especially strong. Osler's method in imparting information is forceful and attractive, and he may well be accorded a place in the front rank as a teacher. He writes simple, good, and straight-forward English, which makes his book entertaining as well as instructive reading.

In this edition many changes have been made in order that its teachings may conform to the present state of science. The typhoid fever section has been rewritten in its essentials, the chapter on malarial fever has been recast to include the observations of physicians in the tropics on the subject, and the newer methods of transmission have been set forth. We may mention other diseases by name only in relation to which there is much new material—dysentery, yellow fever, pneumonia, bubonic plague, diphtheria, smallpox, rheumatic fever; these and many others contain the latest additions to our knowledge. Besides, many new articles are presented, so that every essential point in the practice of medicine may be found appropriately covered. It is a treatise that deserves the continued confidence of the undergraduate as well as the practitioner of medicine.

---

THE PATHOLOGY AND TREATMENT OF SEXUAL IMPOTENCE. By VICTOR G. VECKI, M. D. Third edition, revised and enlarged. Duodecimo, 329 pages. Philadelphia and London: W. B. Saunders & Co. 1901. [Price, cloth, $2.00 net.]

This monograph has taken its place as an accepted authority on the subject of impotence. It is not an easy task to treat it adequately and keep within the limits of scientific inquiry. This author has performed the duty with delicacy and thoroughness. This new edition contains such additions as experience has deemed essential to keep it well forward in its teachings, and it will serve to prevent sufferers with this most unfortunate condition from falling into the hands of advertising fakirs. It at least ought to stimulate scientific work in this disorder among physicians, who, perhaps, have been too indifferent on the subject, or possibly, have not regarded it with that seriousness which it deserved. In this edition the subject of treatment has received increased attention, and is as complete as possible.

---

UTERINE FIBROMYOMATA. Their Pathology, Diagnosis and Treatment. By E. STANMORE BISHOP, F. R. C. S., Eng., President Manchester Clinical Society; Fellow of the British Gynecological Society, etc. Octavo, pp. 323; 49 illustrations. Philadelphia: P. Blakiston's Son & Co. 1901. [Price, $3.50.]

This work, although by a writer comparatively unknown, is a monograph of great value and will prove of much service in the study of the uterine fibromata. It is written in a way that convinces one that the author is a man of experience in this field, that he has made a careful study of the subject, and that he does not propose to give advice based upon inadequate knowledge.

The literature of this subject has increased very much within the last few years, indeed it may be said that twelve or fifteen years ago there was nothing very satisfactory in print pertaining to it. Then began the publication in the journals of the cases, singly and in groups of individual operators, but these were so scattered that it was scarcely possible to search for them with satisfaction in the time usually at command for such purposes. Bishop has met this defect most admirably by grouping and crystallising the material of value, so as to make it readily available; foot-note references being used to point out the sources of information.

This author, as might be anticipated, places very little reliance on electricity in the treatment of these neoplasms, except in inoperable conditions, and then he appears to realise that it is only of transitory benefit. The chapter on the technic of operative methods is one of absorbing interest. He divides this portion of his treatise into three main classes: (*a*) methods which decrease the nutrition of the tumor; (*b*) methods which remove the tumor alone; and (*c*) methods which remove the uterus and tumor. He then proceeds to describe each in the clearest possible manner.

We have alluded to Bishop's familiarity with the literature of fibromyomata and his elaborate foot-note references, but we miss allusion to Ill's recent admirable contribution to the subject (*American Medical Quarterly*, April, 1900,) which is one of the best papers lately published on this side of the water. Everywhere this book will be welcomed as a safe guide in the management of these growths.

---

MANUAL OF THE DISEASES OF THE EYE. For Students and General Practitioners. By CHARLES H. MAY, M. D., Chief of Clinic and Instructor in Ophthalmology, Eye Department, College of Physicians and Surgeons, New York. Second edition, revised. 12mo, pp. 421. 275 original illustrations, including 36 colored figures. New York: William Wood & Co. 1901.

This manual has proved its popularity so quickly that a new edition is called for within the first year of its publication. This comes from the practical methods of its author and the excellent descriptions he gives of all the details of ophthalmic work. He has made some changes in a few of the subjects, and added a number of illustrations in the text, together with seven plates. These enhance the value of the book to a considerable degree. Especial attention may be directed with propriety to the chapter on ocular therapeutics, and to the unusually complete index which is also useful as a dictionary of ophthalmologic terms.

---

A HANDBOOK OF PATHOLOGICAL ANATOMY AND HISTOLOGY. With an Introductory Section on Post Mortem Examinations and the Methods of Preserving and Examining Diseased Tissues. By FRANCIS DELAFIELD, M. D., LL. D., Professor of the Practice of Medicine, College of Physicians and Surgeons, Columbia University, New York; and T. MITCHELL PRUDDEN, M. D., LL. D., Professor of Pathology, College of Physicians and Surgeons, New York. Sixth edition. With 13 full-page plates and 453 illustrations in the text in black and colors. Royal 8vo, pp. 839. New York: William Wood & Co. 1901. [Price, muslin, $5.00 net; sheep, $5.75 net.]

This standard work presents itself in its sixth edition for the approval of the medical profession as well as undergraduates. The first part, consisting of three chapters, is taken up with the consideration of post-mortem examinations. Every junior member of the profession should familiarise himself with this subject. He will find nowhere else more complete instruction relating to it.

In the second part are eleven chapters treating of general pathology, in which will be found descriptions of the lesions of acute infectious diseases and their microörganisms; degeneration and inflammation; the histology of tumors; and the lesions induced by poisoning and violent deaths. In the third part, devoted to the subject of special pathology, are fourteen chapters descriptive of the lesions of the several tissues and organs of the body.

The general arrangement of the work is quite the same as heretofore, but much new material has been incorporated in the text, a good deal has been rewritten, many new illustrations have been added, and the whole treatise advanced to the fore-front of latest pathological knowledge. The chapters on the blood are to be commended in particular, and the whole treatise is one of exceptional merit, deserving the high place it has won for itself in the esteem of students and practitioners of medicine in all parts of the world.

---

The Estivo-Autumnal (remittent) Malarial Fevers. By Charles F. Craig, M. D. (Yale), Acting Assistant Surgeon, U. S. Army; Pathologist and Bacteriologist to the U. S. General Hospital, Presidio of San Francisco, Cal.; late Director of the Bacteriological Laboratories of the Sternberg U. S. A. General Hospital, Chickamauga Park, Ga., etc. Octavo, pp. 231. Illustrated by 2 colored plates and 21 clinical charts. New York: William Wood & Co. 1901.

With the new possessions we have lately acquired come new responsibilities, not the least of which is the study of tropical diseases, with a view to their abatement by improved sanitation or their successful treatment, one or both, according to environment and other controlling conditions.

The malarial fevers, of the tropics, especially the estivo-autumnal or remittent type, are singularly fatal in their nature as this author points out, and most active measures are needed to arrest their progress. Not until recently have their parasites been described and their etiology thus established. Craig has made a careful study of the whole subject, and has placed much valuable information in the hands of the profession through the publication of this book.

Mistakes in diagnosis are of frequent occurrence and it is by no means an easy matter to differentially diagnosticate the estivo-autumnal fevers. It is only through the careful study of the various types by skilful observers like Craig, that a satisfactory basis of judgment can be obtained. Every student of the subject should carefully examine this work, which is by far the best exposition of these fevers that has yet been published.

---

A Text-Book of the Practice of Medicine. By James M. Anders, M. D., Ph. D., LL. D., Professor of the Practice of Medicine and of Clinical Medicine, Medico-Chirurgical College, Philadelphia. Fifth edition, thoroughly revised. One handsome 8vo volume of 1,297 pages, fully illustrated. Philadelphia and London: W. B. Saunders & Co. 1901. [Price, cloth, $5.50 net.]

For the fifth time in four years we are called upon to notice Ander's practice. From this it can be seen that it is easily the most popular text-book on the practice of medicine that is at present before the American profession. We have given our views in detail of the scope and plan of the treatise in former reviews and notices, and we feel justified in repeating and reaffirming our previously expressed good opinions.

There has been no change in the original purpose of the author, which was to make his book so practical that it could not fail to be of service to the everyday practitioner, and, withal, scientific enough to meet the demands of the clinical teacher. There is, therefore, less of theory, and more of practical utility in this work than in most text-books on practice.

In this new edition the author has recast much of his previous work on the infectious diseases and has rewritten in large part the sections relating to the transmission of malaria and yellow fever. The role of the mosquito in these diseases is alluded to and many other interesting points are developed. Some new subjects are added, notably those on fatty infiltration of the heart, streptococcus pneumonia, and acute diffuse interstitial nephritis. The entire book has been thoroughly overhauled and brought into perfect harmony with the present day conditions relating to the practice of medicine.

---

SAUNDERS'S QUESTION COMPENDS. Essentials of Refraction and Diseases of the Eye. By EDWARD JACKSON, A. M., M. D., Emeritus Professor of Diseases of the Eye in the Philadelphia Polyclinic. Third edition, revised and enlarged. Duodecimo, 261 pages, 82 illustrations. Philadelphia and London: W. B. Saunders & Co. 1901. [Price, cloth, $1.00 net.]

This author is an experienced teacher, hence knows what the student of refraction most needs. The term student is not intended here to mean undergraduate, but any person who attempts to acquire the scientific knowledge necessary to practise refraction. The book has been increased in size and scope in this edition and now embraces, *en résumé*, everything necessary in such a work. Since question compends appear to be a necessity, let us have good ones like this book.

---

A PRACTICAL TREATISE ON DISEASES OF THE SKIN. By JOHN V. SHOEMAKER, M. D., LL. D., Professor of Skin and Venereal Diseases in the Medico Chirurgical College and Hospital, Philadelphia; Physician to the Philadelphia Hospital for Diseases of the Skin, etc. Octavo, pp. 905. Fourth edition, revised and enlarged. New York: D. Appleton & Co. 1901. [Price, cloth, $5.00; sheep, $6.00.]

The last previous edition of this well established treatise appeared in 1897, and since that time many new facts have been established relating to certain diseases of the skin, hence a new edition becomes important in order that this experienced author may keep his book quite in touch with the period. Dermatology has progressed quite as much as any other department of medical science.

In the present edition the most recent discoveries or observations relating to the etiology, pathology, pathogenesis, diagnosis and treatment of cutaneous diseases are set forth with that excellence of detail which only the experienced teacher can unfold.

An interesting study is the influence of parasites in exciting certain maladies, and Shoemaker has made some original observations on many phases of this subject. He has also paid great attention to treatment, and a very complete formulary, at the end of the book, makes it of special value to the general physician. It is a standard treatise that will continue to maintain its claim to popular favor.

---

PATHOLOGICAL TECHNIQUE. A Practical Manual for Workers in Pathological Histology, including Directions for the Performance of Autopsies and for Clinical Diagnosis by Laboratory Methods. By FRANK P. MALLORY, A. M., M. D., Associate Professor of Pathology, Harvard University Medical School; and JAMES H. WRIGHT, A. M., M. D , Instructor in Pathology, Harvard University Medical School. Second edition, revised and enlarged. Octavo, 432 pages, with 137 illustrations. Philadelphia and London: W. B. Saunders & Co. 1901. [Price, Cloth, $3.00 net.]

Laboratory students and workers of every grade—undergraduates, practicing physicians or pathologists—will be gratified to see the second edition of this excellent manual.

"Among the many changes and additions to this edition may be mentioned the amplification of the description of the parasite of actinomycosis and the insertion of descriptions of the bacillus of bubonic plague, of the parasite of mycetoma, and Wright's methods for the cultivation of anaërobic bacteria. There have also been added new staining methods for elastic tissue by Weigert, for bone by Schmorl, and for connective tissue by Mallory."

Coroners, physicians and official post mortem examiners everywhere cannot be too careful in conducting autopsies. Crime often goes undetected because of the slip-shod way in which the post mortem examiner conducts his work. His duty is imperative—namely, to exhaust every known method to ascertain the cause of death and to lay the best basis for further study by the bacteriologist and histologist in case investigations become necessary on these lines. This book will be vastly helpful to all such officials and should be very carefully studied by them.

The importance of applying laboratory methods to clinical diagnosis is increasing constantly and this work gives clear information on that branch of the subject. It is one of the most valuable books of its class and should be in possession of every laboratory worker.

---

SURGICAL APPLIED ANATOMY. By SIR FREDERICK TREVES, K. C. V. O., C. B., F. R. C. S., Sergeant-Surgeon to the King; Surgeon-in-Ordinary to the Prince of Wales; Consulting Surgeon to the London Hospital, etc. New edition. Revised by the author with the assistance of Arthur Keith, M. D., F. R. C. S.. in one 12mo volume of 576 pages, with 80 engravings. Philadelphia and New York: Lea Brothers & Co., Publishers. 1901. [Price, cloth, $2.00 net.]

The distinguished author of this valuable little work said in the preface to the first edition, that it should be one object of applied anatomy to invest the hard, unassimilable facts of human anatomy with the interest derived from an association with the circumstances of daily life; in other words, said he, it should make the dry-bones live. Sir Frederick has so nearly accomplished the task he set for himself that very little need be added to make it complete. The work is a very great aid to the surgeon, though its original design was to assist students preparing for their final examinations.

Like most new editions this one has been carefully revised, some of it rewritten and considerable new material added. The book is all that could be desired in such a work.

---

MANUAL OF CHEMISTRY. A Guide to Lectures and Laboratory Work for Beginners in Chemistry. Specially adapted for students of medicine, pharmacy and dentistry. By W. SIMON, Ph. D., M. D., Professor of Chemistry in the College of Physicians and Surgeons of Baltimore, in the Maryland College of Pharmacy, and in the Baltimore College of Dental Surgery. Seventh edition, thoroughly revised and much enlarged. Octavo, 613 pages, with 66 engravings, 1 colored spectra plate and 8 colored plates representing 64 of the most important chemical reactions. Philadelphia and New York: Lea Brothers & Co. 1901. [Price, cloth, $3.00 net.]

The subject of chemistry is, speaking generally, a dread to medical students, particularly so when they appear for degree or license examinations. Simon has done much to smooth the rugged pathway for all who feel that the study of this charming science is laborious. Especially does this remark apply to the sections on chemical physics and physiological chemistry. These parts have been very much enlarged, and are now attractive and complete.

The original division of the work into seven parts is adhered to, and these are: (1) chemical physics; (2) principles of chemistry; (3) non-metals and there combinations; (4) metals and their combinations; (5) analytical chemistry; (6) carbon compounds or organic chemistry; and (7) physiological chemistry. The illustrations and plates are of a character to lend aid to a better understanding of the text. The decimal system is observed throughout, and the whole work is such as to elicit our warm commendation.

THE MEDICAL DIRECTORY OF NEW YORK, NEW JERSEY AND CONNECTICUT. Published by the New York State Medical Association. Volume III. 1901.

This book contains, according to the statement of the publication committee, a total of 12,641 names of physicians, of which 10,112 are in the State of New York, 1,472 in New Jersey and 1,060 in Connecticut. It is a well-arranged book of reference and is a credit to those engaged in its preparation.

---

TRANSACTIONS of the Medical Society of the State of New York at its Ninety-fifth Annual Session, held at Albany, January 29, 30 and 31, 1901. Frederic C. Curtis, M. D., Secretary. Published by the Society. 1901.

This valuable book appears again in its usual form. It is pertinent to say a word as to the make-up of the volume. It is standard octavo in size, printed on fine white antique paper, with good plain letter-press, and is bound in green muslin boards with gilt top. It is a model of simplicity and neatness combined with durability.

Not the least important part of the book is the preliminary matter in which occur the president's inaugural address, the reports of the various committees,—publication, legislation, hygiene, prize essays, and the state board of medical examiners, —and various other items, statements, and specifications of interest. These are frequently overlooked by the reader, but they possess importance for the member who should be familiar with the work of the society.

The president chose for the subject of his anniversary address —The radical cure of inguinal hernia, which is an able paper and is copiously illustrated. The volume is full of interesting and instructive material which deserves careful reading by every one fortunate enough to possess the book.

---

TWENTY-SEVENTH ANNUAL REPORT of the State Board of Health of the State of Michigan for the Fiscal Year Ending June 30, 1899. HENRY B. BAKER, M. D., Secretary. 1901.

TWENTY-EIGHTH ANNUAL REPORT of the State Board of Health of the State of Michigan for the Fiscal Year Ending June 30, 1900. HENRY B. BAKER, M. D., Secretary. 1901.

These two reports cover the period between July 1, 1898, and, July 1, 1900, in the operations of one of our most efficient state boards of health. Dr. Baker has long been recognised as authority on public sanitation and hygiene and is one of the most famous executive officers of a health board in the country. His work is quoted in the magazines and in the more permanent literature of state medicine. These two books bear evidence of continued efficiency and painstaking accuracy on the part of the distinguished secretary.

---

TRANSACTIONS of the Medical Association of Central New York, at its Thirty-third Annual Meeting, held at Rochester, October 16, 1900. Vol. VII. Publication Committee, CHARLES A VAN DER BEEK, S. L. ELSNER, WILLIAM C. KRAUSS. Buffalo Medical Journal Print. 1901.

Again the transactions of this active association are replete with interesting and useful information. The work of a single day by any medical body seldom contains so much of real value. The association has strengthened itself since it commenced the publication of its transactions in book form and it should continue the practice.

---

TRANSACTIONS of the Twenty-second Annual Meeting of the American Laryngological Association, held in the City of Washington, D. C., May 1, 2 and 3, 1900. Dr. James E. Newcomb, Secretary. New York: Carey Printing Co. 1901.

This volume, as is usual with the transactions of this association, contains some of the best thought of the year on diseases and accidents of the upper air tract. The members are experienced specialists and their work is entitled to and does receive the attention of the entire medical profession.

